# Prevalence of Temporomandibular Disorders Based on a Shortened Symptom Questionnaire of the Diagnostic Criteria for Temporomandibular Disorders and Its Screening Reliability for Children and Adolescents Aged 7–14 Years

**DOI:** 10.3390/jcm12124109

**Published:** 2023-06-17

**Authors:** Mathias Rentsch, Aleksandra Zumbrunn Wojczyńska, Luigi M. Gallo, Vera Colombo

**Affiliations:** 1 Clinic of Masticatory Disorders, Center of Dental Medicine, University of Zurich, 8032 Zurich, Switzerland; mathias.rentsch@uzh.ch (M.R.); aleksandra.zumbrunn@zzm.uzh.ch (A.Z.W.); luigi.gallo@zzm.uzh.ch (L.M.G.); 2 Public-School Dental Services of the City of Zurich, 8002 Zurich, Switzerland

**Keywords:** adolescents, children, diagnostic criteria for temporomandibular disorders, temporomandibular joint disorders

## Abstract

The prevalence and adequacy of diagnostic approaches for temporomandibular disorders (TMD) in children and adolescents are still matters of debate. This study aimed to determine the prevalence of TMD and oral habits in children and adolescents aged 7–14 years and evaluate the consistency between self-reported TMD symptoms and clinical findings using a shortened Axis I of Diagnostic Criteria for TMD (DC/TMD). Children (aged 7–10) and adolescents (aged 11–14) of both sexes were invited to participate in this study (*n* = 1468). Descriptive statistics for all observed variables and Mann–Whitney U-Tests for the clinical examination were performed. A total of 239 subjects participated in the study (response rate 16.3%). The self-reported prevalence of TMD was found to be 18.8%. The most frequently reported oral habit was nail biting (37.7%), followed by clenching (32.2%) and grinding (25.5%). Self-reported headache increased with age, while clenching and grinding decreased. Based on the answers to the DC/TMD Symptom Questionnaire, subgroups of asymptomatic and symptomatic participants (*n* = 59; 24.7%) were established and randomly selected for the clinical examination (f = 30). The shortened Symptom Questionnaire showed a sensitivity of 0.556 and a specificity of 0.719 for pain during the clinical examination. Although the Symptom Questionnaire exhibited high specificity (0.933), its sensitivity (0.286) for temporomandibular joint sounds was low. Disc displacement with reduction (10.2%) and myalgia (6.8%) were the most common diagnoses. In conclusion, the self-reported prevalence of TMD in children and adolescents in this study was comparable to that reported in the literature for adults. However, the accuracy of the shortened Symptom Questionnaire as a screening tool for TMD-related pain and jaw sounds in children and adolescents was found to be low.

## 1. Introduction

Temporomandibular disorder (TMD) is a collective term describing dysfunction and pain in the masticatory muscles, as well as in the temporomandibular joints (TMJs) and related structures [1]. Subjects with TMD often exhibit a limited range of motion, joint noises, pain, or a combination of these symptoms [2,3]. TMDs are believed to have a complex, multifactorial etiology. According to recent literature, macrotraumas caused by impact injuries to the chin resulting from accidents [4,5,6], as well as microtraumas due to oral parafunctional habits such as clenching or bruxism [7], are considered etiological factors. Additionally, multiple or frequent oral parafunctions are found to be associated with the incidence of TMD [8]. Psychosocial factors such as stress, anxiety, insomnia, and depression [9,10,11,12] can significantly contribute to the development of TMD. For example, anxiety and stress can lead to increased muscle tension, central sensitization, and bruxism, while also reducing coping strategies [12,13,14]. Furthermore, incorporating psychosocial factors into the treatment strategy has been shown to improve the outcome of TMD treatment [15,16]. Systemic diseases such as rheumatoid arthritis, lupus erythematosus, juvenile idiopathic arthritis, and psoriatic arthritis [17] are also involved in the development of TMD. Comorbid pain, including pre-existing lower back pain or genital pain conditions, sleep disturbance or smoking, has been identified as an important predictor for TMD [18].

TMD prevalence in adults is estimated to be in the range of 5–50% [1,19,20]. TMD may occur at any age; however, the peak occurrence is between 20 and 40 years of age [19], and women are approximately twice as likely to be affected than men [21,22]. Furthermore, the congruence between self-reported TMD symptoms and diagnosed TMD shows a sensitivity of 0.43–0.49 and a specificity of 0.93–0.95 [23,24]. Reported TMD prevalence strongly depends on diagnostic criteria, clinical examination protocols, study population, and training of the investigators [21,25,26]. The Research Diagnostic Criteria for Temporomandibular Disorders (RDC/TMD) clinical examination tool, published in 1992 [27], became a gold standard in the diagnosis of TMD and was used for adults but also for adolescents and children with good reliability [28] until the Diagnostic Criteria for Temporomandibular Disorders (DC/TMD) clinical examination protocol replaced the RDC/TMD guidelines in 2014. DC/TMD is a validated screening tool for detecting TMD as well as for differentiating common pain-related TMDs in adults [29]. However, the DC/TMD examination has not yet been validated for use in children and adolescents.

The prevalence of TMD in children has already been investigated in previous studies using different diagnostic systems and has been shown to be similar to that in adults [30,31,32]. In contrast to adults, mixed results on sex differences [33,34,35,36,37] were obtained, and no differences in the mandibular range of motion for children with and without TMD were found [38,39]. Oral habits (i.e., biting nails, clenching or grinding teeth) in children and adolescents were as prevalent as in adults [34,40,41]. However, only a few studies were based on the DC/TMD protocol [33,36,42,43]. Only one study evaluated the accuracy of the DC/TMD protocol for TMD diagnosis in children aged 8–12 years, which showed a lower accuracy than in adults [28]. Lately, an international group of TMD experts tried to find a consensus on the DC/TMD Axis I using a Delphi study. It was agreed appropriate questionnaires about general health and demographics should be created for children (<10 years old) and adolescents (between 10 and 19 years old). Three screening questions (3Q/TMD) [44] instead of the TMD pain screener [45] should be used for both age groups. The Symptom Questionnaire should be rephrased and adapted for each group. Recommended revisions of the clinical examination contained the abandonment of mandatory commands, a different number of palpation sites, and a reduced threshold for limited mouth opening [46]. 

The main objective of this observational study was to conduct a clinical quantitative assessment of representative parameters for TMD in a sample of children and adolescents between the ages of 7 and 14 to evaluate the reliability of a shortened DC/TMD Symptom Questionnaire as a screening tool for clinical examination. The secondary objective was to estimate the self-reported prevalence of TMD in this specific age group using the collected data. 

## 2. Materials and Methods

### 2.1. Recruitment and Study Participants

The city of Zurich, Switzerland, offers a yearly dental check-up free of charge for all children and adolescents between 2 and 18 years of age. Whole school classes attend one of the six public school dental clinics depending on their location. Recruitment and examinations in the present study were conducted in Zurich between August 2019 and February 2020. This study involved children (aged 7–10) and adolescents (aged 11–14) and was performed in one of the public school dental clinics of the city of Zurich.

For recruitment, two to three weeks prior to the visit to the dental clinic, second-, fourth-, sixth-, and eighth-grade teachers were given envelopes to distribute to the pupils. Each envelope contained the study information, an informed consent form, and a questionnaire about TMD symptoms. Pupils took the envelopes home to decide on participation in accordance with their legal guardians. Teachers were instructed to collect the envelopes and return them to the dental clinic on the day of the visit.

**Figure 1:**
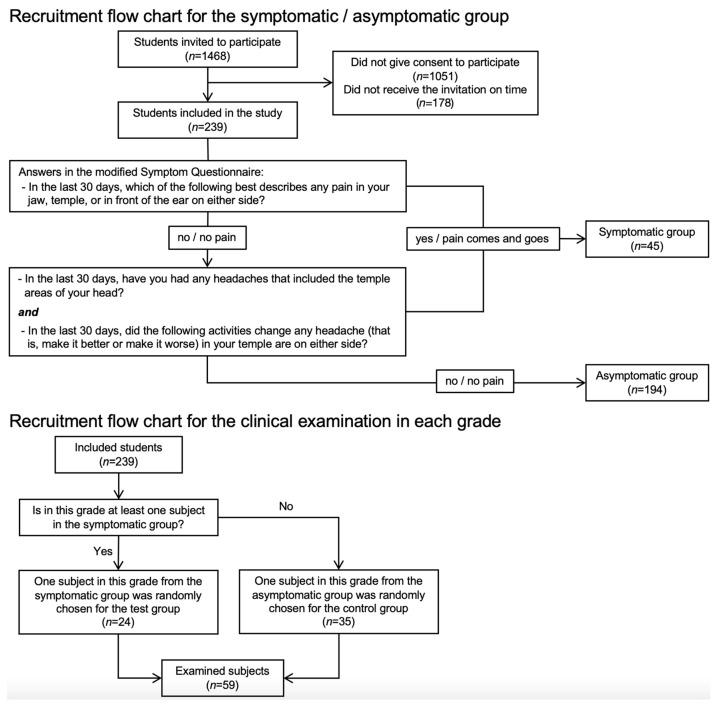
Recruitment flow chart for the test/control sample and for the clinical examination in each class.

On examination day, two groups were created based on the answers given to the questionnaires. The symptomatic group included subjects with any pain in the jaw, temple, anteriorly to or inside the ear, and/or headache that included the temporal areas, and activities that influenced any headache they had experienced in the last 30 days. The remaining subjects (including subjects with pain-free joint noises) were assigned to the asymptomatic group (Figure 1). Afterward, only one child or adolescent of a class was clinically examined after the normal dental check-up. First, a symptomatic participant was randomly chosen for the test group. If the symptomatic participant was missing in the class, an asymptomatic participant was randomly chosen for the control group (Figure 1).

### 2.2. The DC/TMD Symptom Questionnaire

A shortened German version of the DC/TMD questionnaire (Table 1) was used to evaluate the presence of TMD signs or symptoms [47]. All main questions about pain, headache, jaw joint noises, and closed as well as open locking of the jaw were included unaltered. Six questions were skipped due to ease and understanding. In order to evaluate the prevalence of oral habits, five additional questions about oral habits were added. The shortened DC/TMD Symptom Questionnaire and the answers were discussed with the participants prior to the clinical examination to detect divergent answers.

### 2.3. Clinical Examiner (Calibration)

The examiner (MR, pediatric dentist employed by the school dental clinic) underwent training by an orofacial pain specialist with expertise in DC/TMD examination at the University of Zurich (AZW). The training included theory and palpation techniques first on adults and then on an eight-year-old boy at the University of Zurich.

### 2.4. The Clinical DC/TMD Examination

The clinical examination was performed according to the DC/TMD Examination Protocol. Maximum assisted opening, termination of movement, and the examination of supplemental muscle pain with palpation were skipped to ease and avoid discomfort (Table 1). Incisal relationships, maximum opening, and jaw movements were measured with a ruler. TMJ noises were detected bilaterally via palpation. Calibration of the palpation pressure was conducted using commercially available electronic scales [48]. The use of mandatory DC/TMD commands was omitted due to unimpaired results [49] and better understanding for children and adolescents.

### 2.5. Diagnosis of TMD

TMD diagnosis was made based on the DC/TMD Diagnostic Decision Tree and, accordingly, the Diagnostic Criteria Table. The shortened DC/TMD Symptom Questionnaire (Table 2) and the condensed DC/TMD clinical examination were incorporated into the TMD diagnosis. The diagnoses of intra-articular joint disorders were made based on the clinical findings.

### 2.6. Data Analysis and Statistics

The questionnaire was analyzed with descriptive statistics using crosstabulations (e.g., age group, sex). The data were summarized and visualized with tables and diagrams. In a further step, Pearson’s correlation coefficients were used to correlate the data of the questionnaire with sex, age group, and test and control sample. Results of the clinical examination were analyzed with descriptive statistics. Mean values and standard deviations were obtained for all parameters in all conditions observed. Nonparametric Mann–Whitney tests for independent samples were used to assess the differences in the quantitative results of clinical examinations (overjet, overbite, pain-free mouth opening, maximum unassisted mouth opening, laterotrusion, protrusion) between test and control subjects globally in both age groups. If a significant difference was found, pairwise tests for the variable were performed on sex and age group. The level of significance was set at α = 0.05. Microsoft Excel (Version 2019, Microsoft Corporation, Redmond, WA, USA) and SPSS (Version 26.0., IBM Corporation, Armonk, NY, USA) for Windows were used to perform statistical analyses.

## 3. Results

### 3.1. Study Participants

A total of 1468 letters were sent out to 71 classes of four different levels. Of these, 239 children and adolescents (16.3%) agreed to participate in the study. In 8 of the 71 invited classes (178 students), the study envelopes were not delivered in time for the evaluation of potential participants by their teachers. In 5 of 71 classes, none of the participants gave consent to participate. Subjects were divided into two age groups for subsequent analysis: 7–10 years (second and fourth grade, *n* = 137); 11–14 years (sixth and eighth grade, *n* = 102).

### 3.2. Symptom Questionnaire

**Figure 2:**
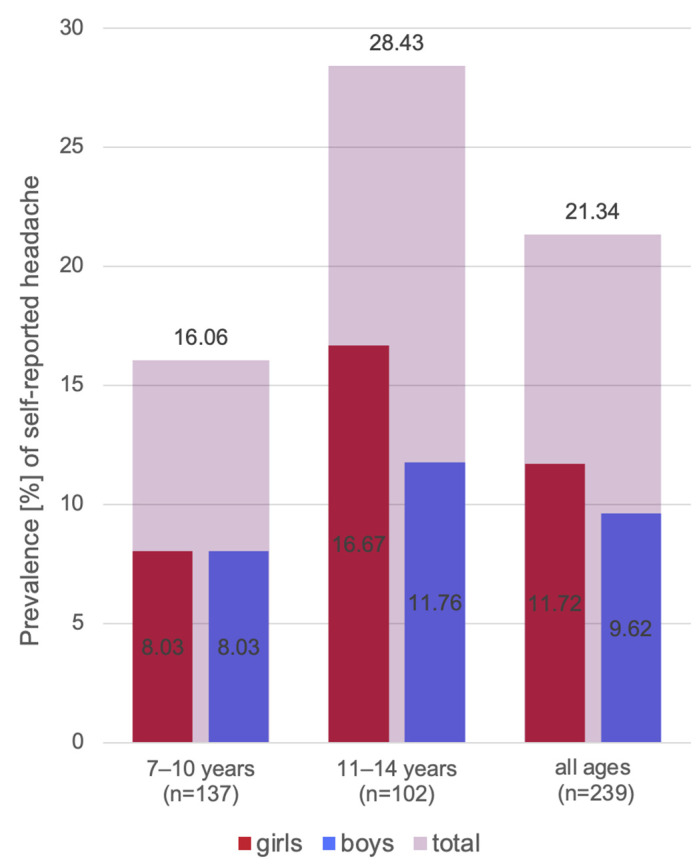
Increase in prevalence of self-reported headache by age and sex.

A total of 239 subjects completed the shortened DC/TMD Symptom Questionnaire. The entire study sample consisted of 114 boys (47.7%) and 125 girls (52.3%). The mean age of all participants was 10.0 ± 1.9 (range 7–14 years). Almost 19% of the participants reported that they were currently suffering from TMD symptoms. Sixty-six subjects (27.6%) stated that they had experienced pain in the jaw, temple, or anteriorly to or inside the ear on either side earlier in their life. In addition, 32 subjects (13.4%) described pain that “comes and goes” in the past 30 days, and jaw activities modified the pain in almost a third of the subjects. Temporal headache in the last 30 days was reported by 51 of 239 children and adolescents (21.3%). In almost 30% of these cases, chewing and/or habits modified the headache. Pearson’s correlation coefficient of 0.944 showed an increase in the prevalence of headage with age (Figure 2).

TMJ noises occurring in the last 30 days were reported by 26 individuals (10.9%). At least one oral habit was found in 160 subjects (66.9%). The most frequently reported oral habit was nail biting, which was found in 90 pupils (37.7%), followed by clenching in 77 pupils (32.2%), and grinding teeth in 61 pupils (25.5%). Self-reported teeth grinding and clenching decreased with age. Detailed information about the answers to the shortened Symptom Questionnaire and oral habits can be found in Table 2.

### 3.3. Clinical Examination

Of the 59 subjects examined clinically, 29 were boys (49.2%) and 30 were girls (50.8%), with a mean age of 10.0 ± 2.3 years (range 7–14). The age distribution of the subjects was as follows: 35 subjects (59.3%) in the younger age group and 24 subjects (40.7%) in the older age group. Of the whole sample, 35 (59.3%) asymptomatic subjects belonged to the controls and 24 (40.7%) symptomatic subjects to the test group. No subject reported changes in the given answers in the Symptom Questionnaire.

#### 3.3.1. Self-Reported Localization of Pain

A total of 14 (23.7%) participants (control: *n* = 0; test: *n* = 14) reported pain in the temporomandibular region. Among them, the masseter muscle was indicated as a site of pain eight times, the temporal muscle four times, and the TMJs eight times. Headache was indicated by 20 (33.9%) pupils (control: *n* = 4; test: *n* = 16). Nine subjects indicated the temporal region (control: *n* = 2; test = 7), ten (control: *n* = 2; test: *n* = 8) indicated other regions, and one test subject reported headache in both the temporal region and other regions. 

#### 3.3.2. Jaw Motion and TMJ Sounds

Both pain-free mouth opening and maximum unassisted mouth opening ranged between 35 and 61 mm. The mean measured values and standard deviation for mouth opening and lateral and protrusive movements divided by age, sex, and control and test sample are shown in Table 3. The Mann–Whitney test revealed significantly larger pain-free mouth opening (*p* = 0.025) and maximum unassisted opening (*p* = 0.015) in the test group than in the control group. Maximum unassisted opening increased significantly with age (*p* = 0.014). Sex did not seem to have an influence on maximum pain-free and unassisted mouth opening. Familiar pain was reported by five children (control: *n* = 2; test: *n* = 3) during opening movements and by three test subjects during horizontal movements.

Jaw joint noises occurred in 14 subjects (23.7%); in six of these cases (control: *n* = 4; test: *n* = 2), a sound was heard by the child and detected by the examiner, and in eight cases (control: *n* = 7; test: *n* = 1), the sound was only detected by the examiner.

#### 3.3.3. Palpation of Muscles and TMJs

Pain during palpation of the masseter and temporal muscle was indicated by 20 subjects (control: *n* = 10; test: *n* = 10). Additionally, three test subjects (12.5%) reported familiar pain, while one control pupil (2.9%) showed familiar pain during muscle palpation. During palpation of the TMJs, 23 children reported pain (control: *n* = 9; test: *n* = 14). In addition, six of them (control: *n* = 1; test: *n* = 5) indicated that they had familiar pain. The most frequently reported site of pain during palpation was the lateral pole of the TMJs.

### 3.4. Comparison between Symptom Questionnaire and Self-Reported Localization of Pain

The 41 subjects examined (control: *n* = 35; test: *n* = 6) who stated that they did not experience episodic pain in the Symptom Questionnaire also reported no pain location prior to the examination. However, four subjects who reported episodic pain in the questionnaire did not indicate any pain location. The remaining 14 test subjects localized pain in both the questionnaire and the examination. 

Three pupils (5.1%) provided divergent answers between the Symptom Questionnaire and self-reported headache localization on examination day, whereas the other responses were consistent.

### 3.5. Comparison of Symptom Questionnaire and Clinical Examination

**Figure 3:**
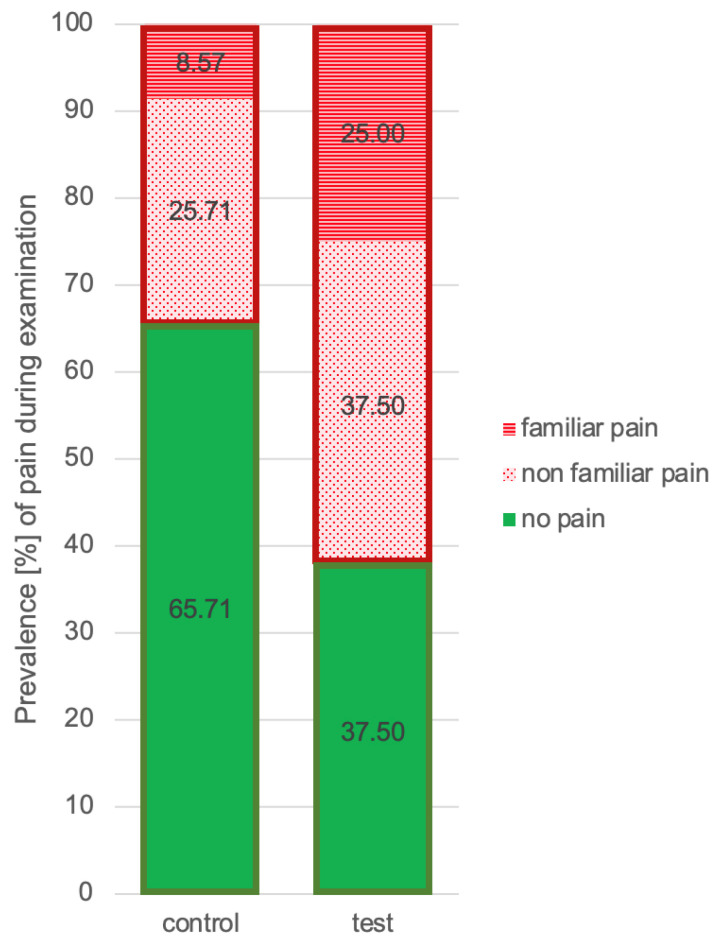
Prevalence of pain during examination (palpation and mouth movements) by control and test.

The sensitivity and specificity for the pain answers in the Symptom Questionnaire and the clinical findings were 0.556 and 0.719. During the clinical examination, 34.3% of the controls and 62.5% of the test subjects reported pain. Pain during palpation of the masseter muscle, temporal muscle, and/or TMJs was recorded in 14 of 24 pupils (58.3%) in the test group. In addition to pain during palpation, six children also showed pain during mandibular movements. Only one child presented pain during the opening phases alone. Furthermore, 12 control subjects (34.3%) reported pain at palpation and 5 (14.3%) during jaw movements. No pain during the clinical examination was found in 23 control subjects and 9 test subjects (Figure 3).

Sensitivity and specificity for joint sounds in the Symptom Questionnaire were 0.286 and 0.933. Subjects reporting joint sounds indeed showed a clicking sound during movement in four out of seven cases. A sound was also recorded in 10 of 52 pupils (19.2%) that did not report experiencing sounds in the questionnaire. Moreover, 8 of these 10 pupils heard no sound during the examination, even when indicated by the examiner.

### 3.6. Comparison between Self-Reported Pain Localization and Clinical Examination

The sensitivity and specificity of positive pain localization were 0.407 and 0.906. A total of 64.4% of the pupils who indicated no pain location also felt no pain during the examination. Agreement between self-reported pain localization and clinical examination was achieved in 81% of cases for the masseter and/or TMJ structures and in 25% for the temporal muscle. Additionally, we combined positive pain localization and temporal headache localization to verify the accuracy of the temporal muscle. As a result, we improved sensitivity but weakened specificity. For the combined measure, we observed a sensitivity and specificity of 0.484 and 0.857.

### 3.7. Diagnoses

Ten individuals had one or more TMD diagnoses. Disc displacement with reduction in five subjects was the most common diagnosis, followed by local myalgia, which was reported in two subjects. Arthralgia was diagnosed in one case, whereas two subjects showed combined diagnoses: in one case local myalgia, arthralgia, and disc displacement with reduction, and in the other myofascial pain with referral combined with arthralgia (Table 4).

## 4. Discussion

### 4.1. Prevalence of Self-Reported Oral Habits and TMD

The aim of the present study was to estimate the prevalence of oral habits, self-reported TMD symptoms, and TMD diagnosed using a shortened German version of the DC/TMD. The most frequently reported oral habit was nail biting, followed by teeth clenching and grinding, both of which decreased with age. Conversely, self-reported headache was more frequent with increasing age. The prevalence of self-reported TMD was comparable to the adult population or even slightly lower. Furthermore, disc displacement with reduction was the most common diagnosis, followed by myalgia.

#### 4.1.1. Oral Habits

Nail biting was found to be the most frequently mentioned oral habit, followed by teeth grinding and clenching, which decreased with age. Our results agree with those of other studies conducted on similar age samples, which showed a prevalence of nail biting around 44–58% and 10–32% for clenching/grinding [34,35,50]. The importance of oral habits for the onset of TMD and the cause-and-effect relationship need to be re-examined in larger longitudinal studies [51,52].

#### 4.1.2. Headache

Our results indicate an increase in headache with age, which corresponds with a nationwide Austrian study that showed that the incidence of headache is associated with older age in pediatric subjects [53]. Furthermore, seven out of ten subjects diagnosed with TMD according to DC/TMD criteria reported suffering from headache in our study. A prospective study showed that the presence of other pain conditions (e.g., headache) at baseline is a predictive factor for the onset of facial pain and TMD in 11-year-olds [54]. Moreover, comorbidity between TMD and headache is bidirectional for both conditions [55]. Therefore, children and adolescents with headache should be screened early for signs of TMD.

#### 4.1.3. Prevalence of Self-Reported TMD and Diagnosis of TMD

The prevalence of pain-related TMD in our study was 18.8%, in agreement with a Swedish systematic review in which the prevalence ranged from 7.3% to 30.4% [30], and with a Dutch cross-sectional questionnaire survey with a prevalence of self-reported TMD of 21.6% [35]. Compared with adults, the prevalence of TMD in children appears to be similar or slightly lower [21,30,56]. The most frequent diagnosis was disc displacement with reduction, followed by myalgia. Even though our results may be compromised because of the shortened Symptom Questionnaire and the clinical examination, they are consistent with other European studies [33,34,50,57]. In contrast, other studies from Brazil, China, and Saudi Arabia found that myofascial pain was the most common diagnosis [2,37,58]. Therefore, ethnic discrepancies cannot be ruled out, as has also been observed in the adult population in the United States [21]. Nevertheless, because of the different forms of clinical examination and different diagnostic criteria, the results are not completely comparable.

### 4.2. Reliability of the DC/TMD Symptom Questionnaire

In addition to TMD prevalence, we analyzed the reliability of a self-completed Symptom Questionnaire as a screening tool in children and adolescents aged 7–14 years. Our main results show low sensitivity and specificity of the Symptom Questionnaire for pain. Compared to the given answers in the Symptom Questionnaire, specificity for pain during the clinical examination increased when the subject was directly asked about the location of pain, while sensitivity decreased. Pain during palpation was mainly found in the lateral TMJ pole or in the masseter muscle, while several controls also reported pain. Most of the subjects who indicated pain in the masseter muscle and/or TMJs also experienced pain during the examination, while for the temporal muscle, this was the case in only a quarter of the subjects. Sensitivity for joint noises was low, while specificity was high. TMJ noises occurred in almost a quarter of the sample, whereas the examiner alone heard sounds in half of the subjects.

#### 4.2.1. DC/TMD Symptom Questionnaire

Our findings show that the reliability of the shortened DC/TMD Symptom Questionnaire in children is lower than for the full version in adults. Nevertheless, our findings are comparable with a German study that used the full version for children aged 8–12 [42]. Based on history and clinical examination, the DC/TMD shows good sensitivity and specificity for TMD in adults [29]. The discrepancy between the two groups could be explained by differences in pain perception [59] and in pain memory [60]. Furthermore, the unequal comprehension and language ability of the subjects due to their development [61] could have led to a misunderstanding of the questions or the clinical examination. 

Therefore, our findings indicate that the shortened DC/TMD Symptom Questionnaire is not an adequate screening tool for TMD in children and adolescents. In conclusion, the TMD pain screener [45] presumably shows similar results because the Symptom Questionnaire includes its items. The three validated screening questions (3Q/TMD) are used in adults and adolescents with good reliability [44,62,63]. Although the screening tool has not yet been tested for children, its use has recently been recommended for both age groups [46]. Further studies are needed to validate the 3Q/TMD in children.

Despite low reliability, we cautiously recommend the use of the DC/TMD assessment protocol as a reference template in general practice. The current DC/TMD protocol can serve as a scheme for dentists to minimize the number of undiagnosed cases until new guidelines and assessment tools are established. The lack of a standardized protocol demonstrates the need for a validated screening and examination tool for children and adolescents. An international consortium of TMD experts is therefore currently working on developing an adapted and validated DC/TMD Axis I and II protocol specifically designed for this particular age group [46,64].

#### 4.2.2. Mouth Opening and Horizontal Movements

The older age group showed significantly larger unassisted mouth opening than the younger age group. Additionally, test subjects also showed significantly larger unassisted mouth opening than controls. A Swiss study measured maximum unassisted mouth opening in 20,719 pupils (F: 10,060 with a median age range of 9.9 years (3.3–18.3); M: 10,659 with a median age range of 10.0 years (2.8–18.7)). Up to the age of 13, no significant sex differences were found. Later, between the ages of 14 and 17, boys showed greater mouth opening than girls. In summary, their study showed an increase in mouth opening with age, but with a wide range within children of the same age group, most likely due to different craniofacial morphologies and skeletal ages. Therefore, they recommended observing individual changes in maximum mouth opening in children at high risk for TMJ afflictions [65]. Our results of greater unassisted mouth opening in the test group could be explained by the wide mouth opening range within children of the same age group and the differences in craniofacial morphology and skeletal age. Not examining maximum assisted opening might negatively affect the sensitivity and specificity for myalgia and arthralgia.

#### 4.2.3. TMJ Noises

More than half of the children with TMJ noises were not aware of any sounds, although they were present during movements, as identified by the examiner. Our findings are consistent with those of a German research group [42] which found that only 3.2% of children were aware of TMJ sounds. Therefore, they suggest that only examiner confirmation should be considered in cases where an examination is needed to diagnose degenerative joint diseases, although patient confirmation is required for the diagnosis [42]. The sensitivity for the diagnosis of disc displacement with reduction in adults is 0.34, while the specificity is 0.92 [29]. Magnetic resonance imaging (MRI) improves sensitivity by up to 78% [66]. Furthermore, a retrospective study of 56 patients with multiple sclerosis without TMD symptoms showed deviated disc position in 12% of cases (Schuknecht B, Kuhn F, MRI Zürich, unpublished normative data 2017). Therefore, inconsistency between self-reported symptoms and clinical signs should be considered when diagnosing disc displacement with reduction in adults and children. 

#### 4.2.4. Palpation of Muscle and TMJs

Pupils in the control and test sample overall reported pain during palpation mainly in the masseter and TMJs, with good congruence between the location of self-reported pain and clinical examination, whereas there seemed to be a discrepancy for the temporal muscle. Therefore, we pooled pain in the temporal muscle and headache in the temporal region to see if we were able to increase sensitivity. The improved results suggest that children and adolescents are unaware of the difference between pain in the temporal region and headache in the temporal region. When defining new questionnaires, this result should be taken into consideration. 

Furthermore, although subjects reported no pain in the questionnaire, almost 30% experienced pain during palpation and a non-negligible number of controls reported familiar pain. Similar results were found in a German study, which showed that 36.2% of the children experienced pain during palpation, although they reported no pain previously [42]. Therefore, the question arises whether the amount of pressure applied is too high or whether new examination questions appropriate for children with higher sensitivity and specificity should be formulated.

### 4.3. Limitations

The main limitation of this study was the use of a shortened version of the existing DC/TMD Symptom Questionnaire and examination protocol, which has not yet been validated for children and adolescents [29]. An international consortium of TMD specialists recently recommended some adjustments to the adult version of the DC/TMD [46]. Although most of the recently published suggestions have already been included in our study, we did not use a modified Symptom Questionnaire adapted for each age group. Instead, we shortened the existing DC/TMD Symptom Questionnaire and examination protocol. Regardless of the alterations, the results should maintain their comparability with other studies, and the impact on the DC/TMD Diagnostic Decision Tree and Table should be minimized. Three of six skipped questions from the DC/TMD Symptom Questionnaire and two of three skipped examinations did not influence the DC/TMD Diagnostic Decision Tree and Table in our study. Intra-articular joint disorders could not be clearly specified in one case (participant number 223) because of the omission of three questions in the Symptom Questionnaire. The other five diagnoses could be correctly made after the DC/TMD Diagnostic Decision Tree and Table. Furthermore, maximum assisted opening was not examined, which might have led to false-negative (undetected) diagnoses concerning myalgia and arthralgia. Therefore, the prevalence of myalgia and arthralgia might be underestimated in our study. However, Katsikogianni et al. showed that there is no significant difference between maximum assisted or unassisted opening in children with or without pain report, and that maximum opening (assisted or unassisted) was painful for 69% of the children with pain and for 41% of the children without pain [42]. Therefore, we decided to omit this examination step to avoid discomfort and additional pain for the pupils although the results may be compromised. Despite our study limitations, our results remain comparable to the current literature [30,33,34,35,50,57].

Another limitation of this study was the number of participants due to the logistics of the study. In addition, there were some potential limitations due to the recruitment strategy. Participants were recruited indirectly by their teachers and depended on them for the delivery of the study documents on the day of the dental visit. Although the differences between children-reported and parent-reported pain were low [67], bias due to parents’ answers in the questionnaire cannot be excluded.

### 4.4. Implications

Early screening and diagnosis are important to avoid misinformation, to improve prevention of chronic pain, and to reduce overtreatment [68,69,70]. Moreover, orofacial pain has an individual but also a social and economic burden [71]. TMD has a strong impact on quality of life [72], and TMD patients consult several healthcare providers before seeing a TMD specialist [73,74]. In addition, treatment costs are estimated at CHF 1778 (approximately USD 1950) per patient in Switzerland, which is almost 30% of the average monthly salary. About 45% of the costs are covered by general insurance, while 55% are borne by the patient [75]. On the other hand, TMD patients show reduced productivity at work [71] and increased school absences [76]. Therefore, early screening and proper diagnosis, together with appropriate treatment, are essential for each individual and society. Dentists could play a key role because they are often the first contact and periodically check their patients through regular recall and follow-up systems. The lack of a standardized screening and diagnostic tool with high accuracy for children complicates the work of the general dentist. We hope that our findings will help improve awareness of the signs and symptoms of TMD in children and adolescents among clinicians and emphasize the need for standardized screening and examination tools. In addition, we hope that our results contain useful information for the creation of a new assessment protocol.

Another important aspect of TMD is its large intra-individual fluctuation during the affected person’s lifetime [77,78], which demonstrates the need for longitudinal studies during childhood and adolescence. Further follow-up studies can better elucidate the development of TMD in relation to age.

## 5. Conclusions

In conclusion, the accuracy of the self-completed shortened DC/TMD Symptom Questionnaire is low. The reason for this is either our adjustments, which may have compromised the diagnostic process and the TMD diagnosis itself, or the fact that neither the Symptom Questionnaire or the clinical examination protocol is suitable for children and adolescents. Standardized screening and examination tools are therefore required for these specific age groups. Dentists should be aware of the relatively high prevalence of TMD in children and adolescents. Furthermore, TMJ sounds are often not perceived by subjects in this age group. The masseter and TMJs show good congruence between self-reported pain location and clinical examination. Children and adolescents tend not to be able to distinguish between temporal pain and temporal headache. Disc displacement with reduction is the most common type of TMD among pediatric subjects, followed by myalgia.

## Figures and Tables

**Table 1:** Comparison between the original DC/TMD protocol [47] and the shortened version.

DC/TMD Symptom Questionnaire		DC/TMD Examination Protocol	
Original Version	Shortened Version	Original Version	Shortened Version
SQ1	SQ1	E1a	E1a
SQ2	—	E1b	E1b
SQ3	SQ3	E2	E2
SQ4	SQ4	E3	E3
SQ5	SQ5	E4a	E4a
SQ6	—	E4b	E4b
SQ7	SQ7	E4c	—
SQ8	SQ8	E4d	—
SQ9	SQ9	E5	E5
SQ10	—	E6	E6
SQ11	—	E7	E7
SQ12	—	E8	E8
SQ13	SQ13	E9	E9
SQ14	—	E10	—
—	OH *		

Numbers indicate the questionnaire number or the examination step. SQ = Symptom Questionnaire; E = examination; OH = oral habits. * Additional questions about oral habits: A. Do you grind your teeth?; B. Do you clench your teeth?; C. Do you bite your nails?; D. Do you suck a pacifier or your thumb?; E. Do you play a wind instrument?

**Table 2:** Prevalence of self-reported pain, jaw function disturbances, and orals habits as reported in the shortened DC/TMD Symptom Questionnaire.

Answers (*n* = 239)	No/No Pain *n* (%)					Yes/Pain Comes and Goes *n* (%)				
	Total	Girls	Boys	Aged 7–10	Aged 11–14	Total	Girls	Boys	Aged 7–10	Aged 11–14
Ever experienced pain in the temporomandibular region	173 (72.4)	92 (73.6%)	81 (71.1)	100 (73.0)	73 (71.6)	66 (27.6)	33 (26.4)	33 (28.9)	37 (27.0)	29 (28.4)
Description of any pain in the temporomandibular region in last 30 days	207 (86.6)	109 (87.2)	98 (86.0)	122 (89.1)	85 (83.3)	32 (13.4)	16 (12.8)	16 (14.0)	15 (10.9)	17 (16.7)
Pain during jaw activities										
A. Chewing	227 (95.0)	119 (95.2)	108 (94.7)	132 (96.4)	95 (93.1)	12 (5.0)	6 (4.8)	6 (5.3)	5 (3.6)	7 (6.9)
B. Opening/movements to the front or to the side	232 (97.1)	122 (97.6)	110 (96.5)	133 (97.1)	99 (97.1)	7 (2.9)	3 (2.4)	4 (3.5)	4 (2.9)	3 (2.9)
C. Jaw habits	229 (95.8)	120 (96.0)	108 (95.6)	136 (99.3)	93 (91.2)	10 (4.2)	5 (4.0)	5 (4.4)	1 (0.7)	9 (8.8)
D. Other jaw activities (talking, kissing, yawning, …)	235 (98.3)	123 (98.4)	112 (98.2)	137 (100)	98 (96.1)	4 (1.7)	2 (1.6)	2 (1.8)	0 (0.0)	4 (3.9)
Temporal headache in the last 30 days	188 (78.7)	97 (77.6)	91 (79.8)	115 (83.9)	73 (71.6)	51 (21.3)	28 (22.4)	23 (20.2)	22 (16.1)	29 (28.4)
Temporal headache during jaw activities										
A. Chewing	223 (93.3)	115 (92.0)	108 (94.7)	132 (96.4)	91 (89.2)	16 (6.7)	10 (8.0)	6 (5.3)	5 (3.6)	11 (10.8)
B. Opening/movements to the front or to the side	232 (97.1)	121 (96.8)	111 (97.4)	133 (97.1)	99 (97.1)	7 (2.9)	4 (3.2)	3 (2.6)	4 (2.9)	3 (2.9)
C. jaw habits	224 (93.7)	115 (91.2)	109 (96.5)	132 (96.4)	92 (90.2)	15 (6.3)	11 (8.8)	4 (3.5)	5 (3.6)	10 (9.8)
D. Other jaw activities (talking, kissing, yawning, …)	228 (95.4)	118 (94.4)	110 (96.5)	134 (97.8	94 (92.2)	11 (4.6)	7 (5.6)	4 (3.5)	3 (2.2)	8 (7.8)
Jaw joint noises	213 (89.1)	109 (87.2)	104 (91.2)	121 (88.3)	92 (90.2)	26 (10.9)	16 (12.8)	10 (8.8)	16 (11.7)	10 (9.8)
Closed locking of the jaw	233 (97.5)	121 (96.8)	112 (98.2)	136 (99.3)	97 (95.1)	6 (2.5)	4 (3.2)	2 (1.8)	1 (0.7)	5 (4.9)
Open locking of the jaw	236 (98.7)	123 (98.4)	113 (99.1)	135 (98.5)	101 (99.0)	3 (1.3)	2 (1.6)	1 (0.9)	2 (1.5)	1 (1.0)
Oral habits										
A. Grinding	162 (67.8)	78 (62.4)	84 (73.7)	85 (62.0)	77 (75.5)	77 (32.2)	47 (37.6)	30 (26.3)	52 (38.0)	25 (24.5)
B. Clenching	178 (74.5)	96 (76.8)	82 (71.9)	101 (73.7)	77 (75.5)	61 (25.5)	29 (23.2)	32 (28.1)	36 (26.3)	25 (24.5)
C. Nail biting	149 (62.3)	82 (65.6)	67 (58.8)	99 (72.3)	50 (49.0)	90 (37.7)	43 (34.4)	47 (41.2)	38 (27.7)	52 (51.0)
D. Pacifier/thumb sucking	231 (96.7)	121 (96.8)	110 (96.5)	131 (95.6)	100 (98)	8 (3.3)	4 (3.2)	4 (3.5)	6 (4.4)	2 (2.0)
E. Wind instrument	212 (88.7)	109 (87.2)	103 (90.4)	122 (89.1)	90 (88.2)	27 (11.3)	16 (12.8)	11 (9.6)	15 (10.9)	12 (11.8)

**Table 3:** Average values measured in millimeters (mm) and standard deviation (*SD*) for mouth opening, lateral movements, and protrusion divided according to age group, sex, and control/test.

Group	Pain-Free Opening	Maximum Un- Assisted Opening	Right Lateral Movement	Left Lateral Movement	Protrusion
Age Group (in Years)					
7–10	45.8 (5.76)	46.71 (5.72)	9 (2.11)	9.31 (1.97)	9.4 (2.14)
11–14	48.29 (5.61)	50.17 (5.47) *	9.54 (1.59)	9.58 (2.54)	10.17 (1.27)
Sex					
Girls	47.07 (5.36)	48.4 (5.46)	8.83 (1.82)	9.4 (1.71)	9.37 (1.87)
Boys	46.55 (6.29)	47.83 (6.27)	9.62 (1.97)	9.45 (2.64)	10.07 (1.83)
Control/Test					
Control	45.49 (6.1)	46.63 (5.96)	9.09 (2.27)	9.11 (2.61)	9.51 (3.67)
Test	48.75 (4.78) *	50.29 (4.99) *	9.42 (1.28)	9.88 (1.33)	10 (1.79)
Total					
Total	46.81 (5.79)	48.12 (5.83)	9.22 (1.92)	9.42 (2.2)	9.71 (1.87)

* *p* < 0.05.

**Table 4:** Individually considered TMD diagnoses according to the DC/TMD diagnostic decision tree among 59 examined children and adolescents.

	Number (%)	Sex		Age Group (Years)	
		Girls	Boys	7–10	11–14
**Myalgia**	4 (6.8)	2	2	1	3
Local myalgia	3 (5.1)	2	1	1	2
Myofascial pain with referral	1 (1.7)		1		1
**Arthralgia**	3 (5.1)	1	2	1	2
**Disc displacement with reduction ^†^**	6 (10.2)	2	4	4	2
Total number of examined symptomatic subjects with a TMD diagnosis	10 * (16.9)				

^†^ For one student, the diagnosis of disc displacement could not be subdivided due to the shortened Symptom Questionnaire; * Multiple diagnoses are possible.

## Data Availability

The data are available upon request from the corresponding author.
